# Dapagliflozin effects on haematocrit, red blood cell count and reticulocytes in insulin-treated patients with type 2 diabetes

**DOI:** 10.1038/s41598-020-78734-z

**Published:** 2020-12-28

**Authors:** Jens Aberle, Markus Menzen, Sebastian M. Schmid, Christoph Terkamp, Elmar Jaeckel, Katja Rohwedder, Markus F. Scheerer, John Xu, Weifeng Tang, Andreas L. Birkenfeld

**Affiliations:** 1 Department for Endocrinology and Diabetology, University Medical Centre Hamburg-Eppendorf, Hamburg, Germany; 2 Division of Internal Medicine, Department of Gastroenterology and Diabetology, Community Hospital Bonn, Bonn, Germany; 3 Institute for Endocrinology and Diabetes, University of Lübeck, Lübeck, Germany; 4 German Centre for Diabetes Research (DZD e.V.), Neuherberg, Germany; 5 Department of Gastroenterology, Hepatology and Endocrinology, Hannover Medical School, Hannover, Germany; 6 AstraZeneca GmbH, Wedel, Germany; 7 AstraZeneca, Gaithersburg, MD USA; 8 Institute of Diabetes Research and Metabolic Diseases of the Helmhotz Zentrum München at Eberhard Karls University Tübingen, Tübingen, Germany; 9 Department of Endocrinology, Diabetology and Nephrology, Eberhard Karls University Tübingen, Otfried-Müller-Straße 10, 72076 Tübingen, Germany; 10 Department of Diabetes, School of Life Course Sciences, King’s College London, London, UK

**Keywords:** Type 2 diabetes, Cardiovascular diseases, Risk factors

## Abstract

Recent studies have shown that high-risk patients with type 2 diabetes mellitus (T2DM) treated with sodium glucose cotransporter 2 (SGLT2) inhibitors have improved cardiovascular (CV) outcomes. In an exploratory analysis of data from the EMPA-REG study, elevations in haematocrit were shown to be strongly associated with beneficial CV effects. As insulin treatment has been shown to be antinatriuretic, with an associated increase in extracellular fluid volume, it is important to confirm whether haematocrit increase is maintained with concomitant insulin therapy. Here, we investigate the effect of the SGLT2 inhibitor dapagliflozin on haematocrit, red blood cell (RBC) counts and reticulocyte levels in high-risk patients with T2DM receiving insulin. A 24-week, double-blinded, randomised, placebo-controlled trial (ClinicalTrials.gov: NCT00673231) was reported previously with extension periods of 24 and 56 weeks (total of 104 weeks). Patients receiving insulin were randomised 1:1:1:1 to placebo or dapagliflozin at 2.5, 5 or 10 mg. Haematocrit, RBC and reticulocyte measurements were conducted during this study, and a longitudinal repeated-measures analysis was performed here to examine change from baseline during treatment. Dapagliflozin treatment in combination with insulin resulted in a dose-dependent increase in haematocrit levels and RBCs over a 104 week period. There was a short-term increase in reticulocyte levels at the start of treatment, which dropped to below baseline after 8 weeks. SGLT2 inhibition with dapagliflozin leads to a sustained increase in haematocrit in patients receiving chronic insulin treatment.

## Introduction

Sodium glucose cotransporter 2 (SGLT2) inhibitors, including empagliflozin, dapagliflozin, and canagliflozin, reduce renal glucose reabsorption and increase urinary glucose excretion, lowering HbA_1c_ levels. Data from several cardiovascular (CV) outcome trials in patients with a high risk of CV events and type 2 diabetes mellitus (T2DM) have shown patients treated with SGLT2 inhibitors have improved CV outcomes^1^. However, the observed reductions in HbA_1c_, body weight, blood pressure or uric acid are generally considered to be inadequate to account for the rapid onset and magnitude of this effect. Several explanations have been suggested, including a switch to utilisation of ketones for energy production as well as direct cardiac effects (reviewed in ^2^). In an exploratory analysis of data from the EMPA-REG trial^3^, haematocrit increase during empagliflozin therapy was strongly associated with beneficial CV outcomes.

Results from the DECLARE TIMI 58 study recently demonstrated that patients with T2DM treated with dapagliflozin had a significantly lower rate of CV death or hospitalisation for heart failure than with placebo^4^. Additionally, results from the DAPA-HF trial, which examined patients with heart failure with a reduced ejection fraction, showed the risk of worsening heart failure or death from CV causes was lower with dapagliflozin^5^. Studies have confirmed that haematocrit is also increased in dapagliflozin-treated patients with T2DM over a 12-week period^6,7^. However, insulin treatment has been reported to have a sustained antinatriuretic effect^8, 9^, resulting in increased extracellular fluid volume^9^ by acting directly on renal tubular sodium transport^8,9^. DeFronzo *et al.* have previously reported a decrease in sodium clearance within 30 min of starting an insulin infusion, reaching a significant reduction after 60 min and declining to around 50% of the control value within two hours^9,10^. Therefore, it is important to understand whether the effect of SGLT2 inhibitors, such as dapagliflozin, on haematocrit is maintained with concomitant insulin therapy.

Previous studies from Wilding et al*.*^11,12^ have demonstrated the long-term efficacy of dapagliflozin over a period of 104 weeks in patients with T2DM receiving concomitant insulin. Dapagliflozin treatment in addition to insulin therapy was shown to result in long-term reductions in mean HbA_1c_, body weight and insulin dose. Here, we evaluate markers of haemoconcentration measured in dapagliflozin-treated patients with T2DM receiving concomitant insulin.

## Methods

This 24-week, double-blinded, randomised, placebo-controlled trial (NCT00673231) was followed by extension periods of 24 and 56 weeks (104 weeks in total). The study was conducted between 30 April 2008 and 12 January 2011 in 126 centres worldwide. Relevant institutional review boards (International coordinating investigator: J.P.H. Wilding, University Hospital Aintree, Liverpool, UK) and independent ethics committees approved the protocol, and each participant gave written, informed consent. The study was performed according to the Declaration of Helsinki and the requirements of Good Clinical Practice.

A detailed description of the study design and analyses has been published previously^11,12^. T2DM patients (808 in total) enrolled in the study were 18–80 years old, had inadequately controlled HbA_1c_ values (7.5–10.5%), were receiving a stable insulin dose of ≥ 30 units/day (at least 8 weeks) with or without up to two antidiabetic drugs (OADs). Patients receiving loop diuretics were excluded. For the initial 48-week period, patients were randomised 1:1:1:1 to receive placebo or dapagliflozin at 2.5, 5 or 10 mg daily. In addition, patients received their normal open-label insulin dose and any OADs. After 48 weeks, patients receiving 5 mg dapagliflozin were switched to 10 mg. The insulin dose was maintained within 10% of baseline unless changes were required to ensure patient wellbeing. Laboratory analyses of haematocrit, red blood cells (RBCs) and reticulocytes was performed as part of this study. A longitudinal repeated-measures analysis, with a model including fixed categorical effects of treatment, week, and treatment-by-week interaction, and also the continuous fixed covariates of baseline measurement and baseline measurement-by-week interaction, was performed for analyses of laboratory tests in terms of change from baseline during treatment. Data from follow-up visits were summarised descriptively.

## Results

**Figure 1:**
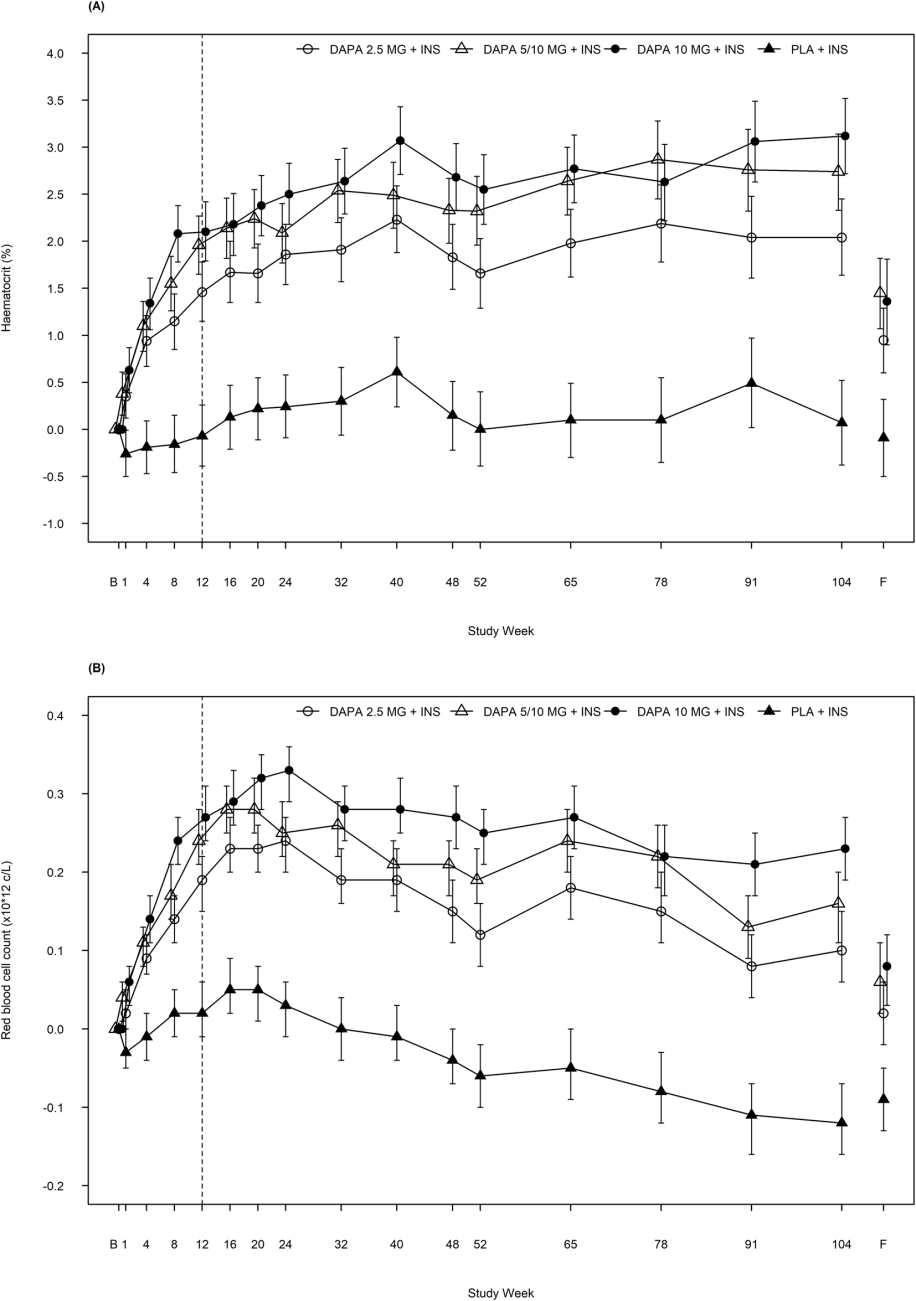

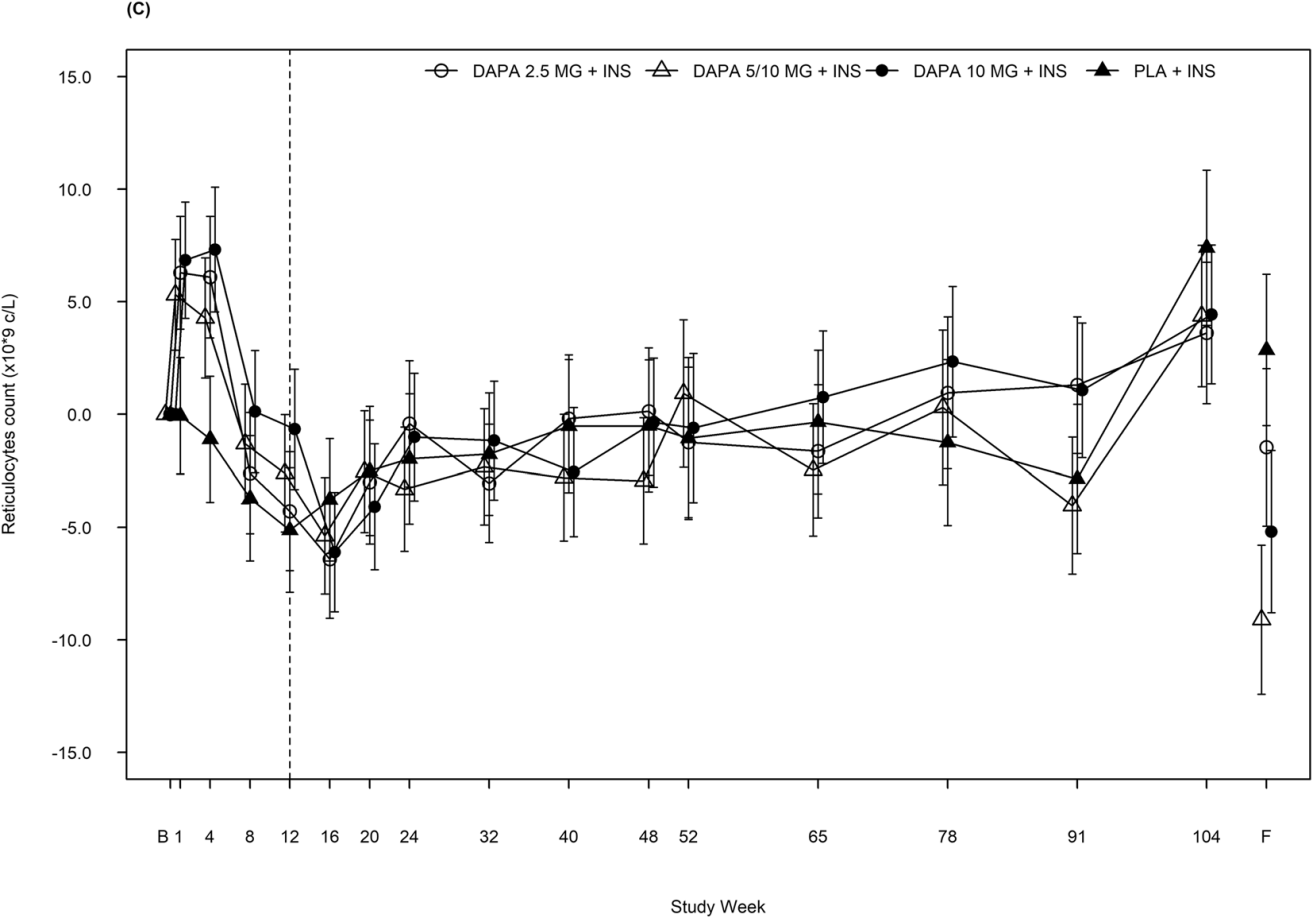
Effect of dapagliflozin on haematocrit, red blood cell and reticulocyte counts. Adjusted mean change from baseline for (**A**) haematocrit, (**B**) red blood cell count and (**C**) reticulocyte count over 104 weeks, including follow-up visit. The vertical dotted line marks the short-term study period (12 weeks). Error bars show ± 95% CI. B, baseline; CI, confidence interval; DAPA, dapagliflozin; F, follow-up; INS, insulin; PLA, placebo.

Baseline characteristics as well as safety and efficacy data have been described previously^11,12^. Changes in haematocrit, RBC and reticulocyte counts in the same study population are shown in Fig. 1 and Table 1.

**Table 1:** Mean change in haematocrit, red blood cell count and reticulocytes from baseline in patients treated with 2.5, 5/10 and 10 mg dapagliflozin and insulin.

	Placebo	Dapagliflozin			
		2.5 mg	5/10 mg	10 mg	Heerspink et al.^7^ 10 mg
*Haematocrit (%)*					
Week 12, n^†^	163	185	188	182	21
Mean (SD)	41.70 (3.60)	42.50 (3.94)	43.21 (3.85)	43.36 (4.08)	43.49 (3.29)
Mean change from BL (SD)	− 0.14 (1.88)	1.57 (2.46)	2.01 (2.17)	2.13 (2.43)	2.15 (2.03)
Adj. mean change from BL (SE)	− 0.07 (0.17)	1.46 (0.16)	1.96 (0.16)	2.10 (0.16)	2.37 (0.54)
95% CI for adj. mean change from BL	(− 0.39, 0,26)	(1.15, 1.78)	(1.65, 2.27)	(1.79, 2.42)	(1.29, 3.46)
Diff. vs. placebo in adj. mean change from BL difference (SE)	–	1.53 (0.23)	2.03 (0.23)	2.17 (0.23)	2.47 (0.75)
95% CI for difference		(1.08, 1.98)	(1.58, 2.47)	(1.72, 2.62)	(0.99, 3.96)
*P* value		< 0.001	< 0.001	< 0.001	0.001
Week 104, n^†^	100	129	124	136	–
Mean (SD)	42.14 (3.80)	43.37 (4.25)	44.49 (4.02)	44.55 (4.25)	
Mean change from BL (SD)	0.12 (2.31)	2.32 (2.57)	2.95 (2.42)	3.06 (2.80)	
Adj. mean change from BL (SE)	0.07 (0.23)	2.04 (0.21)	2.74 (0.21)	3.12 (0.20)	
95% CI for adj. mean change from BL	(− 0.38, 0.52)	(1.64, 2.45)	(2.33, 3.14)	(2.72, 3.52)	
Diff. vs. placebo in adj. mean change from BL difference (SE)	–	1.97 (0.31)	2.67 (0.31)	3.05 (0.30)	
95% CI for difference		(1.37, 2.57)	(2.06, 3.27)	(2.46, 3.65)	
*P* value		< 0.001	< 0.001	< 0.001	
*Red blood cell count (*× *10*^*12*^* c/L)*					
Week 12, n^†^	167	188	190	182	–
Mean (SD)	4.67 (0.41)	4.75 (0.47)	4.87 (0.44)	4.90 (0.43)	
Mean change from BL (SD)	0.02 (0.21)	0.20 (0.28)	0.25 (0.22)	0.28 (0.27)	
Adj. mean change from BL (SE)	0.02 (0.02)	0.19 (0.02)	0.24 (0.02)	0.27 (0.02)	
95% CI for adj. mean change from BL	(− 0.01, 0.06)	(0.15, 0.22)	(0.21, 0.28)	(0.24, 0.31)	
Diff. vs. placebo in adj. mean change from BL difference (SE)	–	0.16 (0.03)	0.22 (0.03)	0.25 (0.03)	
95% CI for difference		(0.12, 0.21)	(0.17, 0.27)	(0.20, 0.30)	
*P* value		< 0.001	< 0.001	< 0.001	
Week 104, n^†^	103	130	128	137	–
Mean (SD)	4.55 (0.40)	4.71 (0.44)	4.81 (0.45)	4.87 (0.44)	
Mean change from BL (SD)	− 0.12 (0.26)	0.14 (0.27)	0.18 (0.27)	0.22 (0.28)	
Adj. mean change from BL (SE)	− 0.12 (0.02)	0.10 (0.02)	0.16 (0.02)	0.23 (0.02)	
95% CI for adj. mean change from BL	(− 0.16, − 0.07)	(0.06, 0.15)	(0.11, 0.20)	(0.19, 0.27)	
Diff. vs. placebo in adj. mean change from BL difference (SE)	–	0.22 (0.03)	0.27 (0.03)	0.34 (0.03)	
95% CI for difference		(0.16, 0.28)	(0.21, 0.34)	(0.28, 0.41)	
*P* value		< 0.001	< 0.001	< 0.001	
*Reticulocytes (*× *10*^*9*^* c/L)*					
Week 4, n^†^	182	197	200	186	24
Mean (SD)	74.77 (26.47)	79.33 (26.33)	80.43 (26.20)	84.18 (27.29)	96.21 (32.26)
Mean change from BL (SD)	− 1.54 (18.09)	7.02 (21.67)	3.69 (21.30)	7.12 (21.71)	11.38^‡^ (27.07)
Adj. mean change from BL (SE)	− 1.10 (1.43)	6.09 (1.38)	4.28 (1.36)	7.32 (1.41)	10.62 (4.29)
95% CI for adj. mean change from BL	(− 3.90, 1.69)	(3.39, 8.79)	(1.62, 6.95)	(4.55, 10.09)	(2.06, 19.17)
Diff. vs. placebo in adj. mean change from BL difference (SE)	–	7.20 (1.98)	5.39 (1.97)	8.42 (2.00)	8.95 (6.06)
95% CI for difference		(3.31, 11.09)	(1.52, 9.26)	(4.49, 12.36)	(− 3.13, 21.04)
*P* value		< 0.001	0.006	< 0.001	0.144
Week 12, n^†^	165	187	189	182	22
Mean (SD)	71.03 (24.94)	68.73 (23.20)	73.78 (25.51)	75.83 (24.35)	79.05 (23.84)
Mean change from BL (SD)	− 5.58 (20.47)	− 2.57 (20.67)	− 3.01 (22.13)	− 1.27 (20.13)	− 3.05^‡^ (27.10)
Adj. mean change from BL (SE)	− 5.13 (1.40)	− 4.29 (1.34)	− 2.61 (1.32)	− 0.66 (1.36)	− 5.81 (3.84)
95% CI for adj. mean change from BL	(− 7.89, − 2.37)	(− 6.92, − 1.66)	(− 5.21, − 0.01)	(− 3.33, 2.00)	(− 13.48. 1.85)
Diff. vs. placebo in adj. mean change from BL difference (SE)	–	0.84 (1.94)	2.52 (1.93)	4.47 (1.95)	− 1.16 (5.27)
95% CI for difference		(− 2.97, 4.66)	(− 1.27, 6.31)	(0.63, 8.30)	(− 11.67, 9.35)
*P* value		0.67	0.19	0.022	0.827
Week 104, n^†^	103	129	127	136	–
Mean (SD)	80.61 (25.81)	76.65 (25.01)	81.50 (23.90)	82.08 (26.69)	
Mean change from BL (SD)	7.91 (20.01)	6.17 (21.84)	2.71 (21.69)	3.38 (21.31)	
Adj. mean change from BL (SE)	7.40 (1.75)	3.62 (1.60)	4.37 (1.60)	4.43 (1.57)	
95% CI for adj. mean change from BL	(3.95, 10.84)	(0.48, 6.76)	(1.23, 7.51)	(1.35, 7.52)	
Diff. vs. placebo in adj. mean change from BL difference (SE)	–	− 3.77 (2.37)	− 3.03 (2.38)	− 2.96 (2.36)	
95% CI for difference		(− 8.43, 0.88)	(− 7.70, 1.64)	(− 7.59, 1.67)	
*P* value		0.112	0.203	0.209	

Mixed model: change from baseline = baseline treatment week*treatment week*baseline.

^†^Number of subjects in safety analysis dataset with non-missing baseline and Week t values.

^‡^Values in Heerspink et al.^7^ reported as × 10^3^ c/µL.

BL, baseline; CI, confidence interval; SD, standard deviation; SE, standard error.

Treatment with dapagliflozin combined with insulin produced a dose-dependent increase in haematocrit compared with placebo and insulin at 12 weeks (Fig. 1A; Table 1). After this initial increase, haematocrit levels remained stable until the end of the 104-week study period (Fig. 1A; Table 1).

RBC counts also increased in a dose-dependent manner after 12 weeks of dapagliflozin treatment (Fig. 1B; Table 1). This was followed by a gradual decline to Week 104, but with counts maintained well above baseline and placebo in the dapagliflozin-treated arms (Fig. 1B; Table 1).

A short-term increase in reticulocyte levels up to Week 4 was observed, followed by a sharp decline to a nadir below the baseline level at Week 12 (Fig. 1C; Table 1). Reticulocyte levels in all treatment arms then remained stable until the end of the 104-week study period (Fig. 1C; Table 1).

## Discussion

SGLT2 inhibitor-dependent increases in haematocrit were previously suggested to be important for reduced CV mortality risk in the EMPA-REG study^3^. Increases in haematocrit have also been observed in patients with T2DM treated with dapagliflozin. For example, Heerspink et al. previously investigated short-term changes in haematocrit, erythropoietin (EPO) and reticulocyte count over 12 weeks of dapagliflozin, hydrochlorothiazide or placebo treatment^7^. After 12 weeks, haematocrit increased in the dapagliflozin group compared with the hydrochlorothiazide and placebo groups. However, in contrast to the current study, these patients did not receive insulin in addition to dapagliflozin treatment.

Insulin is known to have a sustained antinatriuretic effect with concomitant increase in extracellular fluid volume^8,9^. The relationship between haematocrit and insulin sensitivity has previously been studied in healthy volunteers and in patients with non-insulin-dependent diabetes mellitus^13^, indicating that insulin sensitivity is inversely related to haematocrit regardless of glucose tolerance. The authors concluded that blood viscosity may therefore be regulated by insulin resistance or hyperinsulaemia. Because of this decrease in haematocrit observed with insulin, it was unclear whether SGLT2 inhibition could also positively affect markers of haemoconcentration when co-administered with insulin. A significant increase in haemoglobin and haematrocrit with dapagliflozin treatment was reported in a small randomised study in 36 patients with T2DM receiving insulin treatment^14^, suggesting an effect alongside insulin. In our study, this was confirmed with haematocrit and RBC counts showing stable elevation in a dose-dependent manner over 104 weeks, and there was a short-term increase in reticulocyte count. Although EPO was not measured directly here, reticulocyte increase is dependent on EPO.

Elevated haematocrit could be due to reductions in plasma volume caused by SGLT2 inhibitor-related diuresis and natriuresis. Decreased plasma volume has indeed been observed in studies with dapagliflozin-treated patients with T2DM^7,15^. However, it has been noted that diuretic agents appear to be associated with a smaller reduction in CV mortality than was observed in EMPA-REG^3,16^. It is therefore unlikely that simple haemoconcentration is responsible.

Elevated haematocrit could therefore be attributed to increased erythropoiesis. SGLT2 inhibitor treatment has previously been shown to transiently increase EPO levels^6,7^. EPO may be released following SGLT2 inhibition as a response to relative hypoxemia in the renal medulla. Sodium escaping proximal reabsorption may impose a work overload on the distal tubule, resulting in a transient increase in oxygen consumption and reduction in oxygen tension^17^. A recent study reported that dapagliflozin treatment increased erythropoiesis and haematocrit in obese patients with T2DM receiving treatment with metformin (85% of patients), insulin (38%), sulfonylureas (38%) and pioglitazone (21%) for diabetes management^6^. They concluded that this effect was due to mechanisms that involve suppression of hepcidin and modulation of other proteins involved in iron regulation^6^.

Since EPO release induced by SGLT2 inhibitors declines after 4 weeks of treatment^7^, EPO may only be partially responsible for the long-term rise in haematocrit. Our observation that reticulocytes also decrease between weeks 4 and 12 also corroborates this assumption. While increased availability of oxygen carriers may improve oxygen supply to organs and tissues such as the heart and the kidney, administration of EPO in patients with myocardial infarction does not improve outcomes^18^. Therefore, the combination of increasing tissue oxygen delivery mediated by EPO, reducing cardiac pre- and afterload by volume and blood pressure reduction, and the delivery of oxygen-sparing substrates, such as ketones, may be more effective. Moreover, EPO may serve as a beneficial cytokine improving cardiomyocyte mitochondrial function, angiogenesis and inflammation^19^.

Chronic activation of the sympathetic nervous system occurs in patients with T2DM, increasing CV risk (reviewed in ^20^). Resorption of glucose in the proximal tubules mediated by SGLT2 exerts a high renal energy demand, resulting in activation of renal afferent nerves and overactivation of the central sympathetic system and causing sympathetic outflow to the heart, kidneys and vessels^20^. SGLT2 inhibition could reduce glucose reabsorption, reducing renal energy requirements and potentially suppressing signalling of renal afferent nerves to the brain. It is tempting to speculate that these mechanisms, along with SGLT2-induced changes in haemoconcentration, could also contribute to increased CV benefits.

Interestingly, anaemia is common in patients with diabetes and the combination of anaemia, diabetes and renal impairment, the so called cardiorenal-anaemia syndrome, shows particularly negative impact on heart failure prognosis. In line with this notion, the recently published DAPA-HF^21^ trial showed improvement in heart failure outcomes with dapagliflozin^5^. Hopefully, analyses from this study may further elucidate the underlying mechanisms. Additionally, recent results from the EMPEROR trial showed that empagliflozin-treated patients had a lower risk of CV death or hospitalization for heart failure when compared with patients receiving placebo^21^.

While we cannot conclude that the increase in haematocrit observed in our study directly resulted in an improvement in CV outcomes, it is of interest to note that Inzucchi et al*.* found that changes in haematocrit and haemoglobin to be the most important mediators of CV mortality reduction with empagliflozin vs. placebo in their exploratory analysis from the EMPA-REG OUTCOME trial^3^. Further research should clarify the link between such plasma volume markers and CV outcomes.

Finally, we acknowledge the limitations of this clinical study. For example, a control group without insulin treatment would have allowed evaluation of the haematocrit increase with dapagliflozin treatment in the presence vs. absence of insulin. In addition, the insulin exposure evaluated in our study was relatively consistent across study arms. We did not include differences in insulin exposure nor such a control group in our study as such comparisons were not the primary aim of this study. Further studies are required to determine whether the impact of dapagliflozin on haematocrit differs according to different levels of insulin exposure or in patients not receiving concomitant insulin therapy.

## Conclusions

In summary, we show that dapagliflozin induces a long-term and dose-dependent elevation in haematocrit that is preserved in patients receiving concomitant insulin treatment, including chronic insulin therapy. This effect may, in part, be mediated by a concerted time-dependent elevation of EPO, reticulocytes and other, longer term consequences. More research is needed to elucidate other mechanisms contributing to this effect.

## Data Availability

The datasets used and/or analysed during the current study are available from the corresponding author on reasonable request.

## Abbreviations

B: Baseline
CV: Cardiovascular
DAPA: Dapagliflozin
DAPA-HF: Dapagliflozin and Prevention of Adverse Outcomes in Heart Failure
EMPA-REG: Empagliflozin Cardiovascular Outcome Event Trial in Type 2 Diabetes Mellitus Patients-Removing Excess Glucose
DECLARE TIMI 58: Dapagliflozin Effect on Cardiovascular Events–Thrombolysis in Myocardial Infarction 58
EPO: Erythropoietin
F: Follow up
INS: Insulin
OAD: Antidiabetic drug
PLA: Placebo
RBC: Red blood cell
SGLT2: Sodium glucose cotransporter 2
T2DM: Type 2 diabetes mellitus

## References

[1] Zelniker TA (2019). SGLT2 inhibitors for primary and secondary prevention of cardiovascular and renal outcomes in type 2 diabetes: a systematic review and meta-analysis of cardiovascular outcome trials. Lancet.

[2] Verma S, McMurray JJV (2018). SGLT2 inhibitors and mechanisms of cardiovascular benefit: a state-of-the-art review. Diabetologia.

[3] Inzucchi SE (2018). How does empagliflozin reduce cardiovascular mortality? Insights from a mediation analysis of the EMPA-REG OUTCOME trial. Diabetes Care.

[4] Wiviott SD (2019). Dapagliflozin and cardiovascular outcomes in type 2 diabetes. N. Engl. J. Med..

[5] McMurray JJV (2019). Dapagliflozin in patients with heart failure and reduced ejection fraction. N. Engl. J. Med..

[6] Ghanim H (2020). Dapagliflozin suppresses hepcidin and increases erythropoiesis. J. Clin. Endocrinol. Metab..

[7] Lambers Heerspink HJ, de Zeeuw D, Wie L, Leslie B, List J (2013). Dapagliflozin a glucose-regulating drug with diuretic properties in subjects with type 2 diabetes. Diabetes Obes. Metab..

[8] Brands MW (2018). Role of insulin-mediated antinatriuresis in sodium homeostasis and hypertension. Hypertension.

[9] DeFronzo RA (1981). The effect of insulin on renal sodium metabolism. A review with clinical implications. Diabetologia.

[10] DeFronzo RA, Cooke CR, Andres R, Faloona GR, Davis PJ (1975). The effect of insulin on renal handling of sodium, potassium, calcium, and phosphate in man. J. Clin. Investig..

[11] Wilding JP (2014). Dapagliflozin in patients with type 2 diabetes receiving high doses of insulin: efficacy and safety over 2 years. Diabetes Obes. Metab..

[12] Wilding JP (2012). Long-term efficacy of dapagliflozin in patients with type 2 diabetes mellitus receiving high doses of insulin: a randomized trial. Ann. Intern. Med..

[13] Catalano C (1997). Reciprocal association between insulin sensitivity and the haematocrit in man. Eur. J. Clin. Invest..

[14] Nomoto H (2017). A randomized controlled trial comparing the effects of dapagliflozin and DPP-4 inhibitors on glucose variability and metabolic parameters in patients with type 2 diabetes mellitus on insulin. Diabetol. Metab. Syndr..

[15] Dekkers CCJ (2019). Effects of the sodium-glucose co-transporter-2 inhibitor dapagliflozin on estimated plasma volume in patients with type 2 diabetes. Diabetes Obes. Metab..

[16] Olde Engberink RH (2015). Effects of thiazide-type and thiazide-like diuretics on cardiovascular events and mortality: systematic review and meta-analysis. Hypertension.

[17] O'Neill J (2015). Acute SGLT inhibition normalizes O_2_ tension in the renal cortex but causes hypoxia in the renal medulla in anaesthetized control and diabetic rats. Am. J. Physiol. Renal Physiol..

[18] Ali-Hassan-Sayegh S (2015). Administration of erythropoietin in patients with myocardial infarction: does it make sense? An updated and comprehensive meta-analysis and systematic review. Cardiovasc. Revasc. Med..

[19] Mazer, C. D. *et al.* Effect of empagliflozin on erythropoietin levels, iron stores, and red blood cell morphology in patients with type 2 diabetes mellitus and coronary artery disease. *Circulation*. **141**(8), 704–707. 10.1161/CIRCULATIONAHA.119.044235 (2020). 10.1161/CIRCULATIONAHA.119.044235 31707794

[20] Sano M (2018). A new class of drugs for heart failure: SGLT2 inhibitors reduce sympathetic overactivity. J. Cardiol..

[21] Packer, M. *et al.* Cardiovascular and renal outcomes with empagliflozin in heart failure. *N. Engl. J. Med.* **383**(15), 1413–1424. 10.1056/NEJMoa2022190 (2020). 10.1056/NEJMoa2022190 32865377

